# Low-Cycle Fatigue Crack Initiation Simulation and Life Prediction of Powder Superalloy Considering Inclusion-Matrix Interface Debonding

**DOI:** 10.3390/ma14144018

**Published:** 2021-07-18

**Authors:** Shuming Zhang, Yuanming Xu, Hao Fu, Yaowei Wen, Yibing Wang, Xinling Liu

**Affiliations:** 1 School of Aeronautic Science and Engineering, Beihang University, Beijing 100191, China; zhangshuming@buaa.edu.cn (S.Z.); 05640@buaa.edu.cn (Y.X.); SY1905106@buaa.edu.cn (H.F.); 2234389878@buaa.edu.cn (Y.W.); 2 Beijing Key Laboratory of Aeronautical Materials Testing and Evaluation, AECC Beijing Institute of Aeronautical Materials, Beijing 100095, China; liuxinling119@163.com

**Keywords:** powder superalloy, interface debonding, inclusion, life prediction, damage mechanics

## Abstract

From the perspective of damage mechanics, the damage parameters were introduced as the characterizing quantity of the decrease in the mechanical properties of powder superalloy material FGH96 under fatigue loading. By deriving a damage evolution equation, a fatigue life prediction model of powder superalloy containing inclusions was constructed based on damage mechanics. The specimens containing elliptical subsurface inclusions and semielliptical surface inclusions were considered. The CONTA172 and TARGE169 elements of finite element software (ANSYS) were used to simulate the interfacial debonding between the inclusions and matrix, and the interface crack initiation life was calculated. Through finite element modeling, the stress field evolution during the interface debonding was traced by simulation. Finally, the effect of the position and shape size of inclusions on interface debonding was explored.

## 1. Introduction

The turbine disk is the core hot-end component of an aero turbine engine. The temperature in front of the turbine of modern aero engines can be as high as 1800 K and can reach 20,000 revolutions per minute. The complex thermomechanical load leads to high requirements on the strength, reliability, fatigue performance, and creep resistance of engine turbines. Powder superalloys have good oxidation resistance, corrosion resistance, excellent stretching, durability, fatigue performance, and long-term structure stability [1,2,3], and thus they have quickly become the material of choice for the manufacture of high thrust-to-weight ratio engine turbine discs and guide vanes in various countries [4].

However, with extensive use and research, people have gradually realized that powder superalloys also have their own unique problems: thermally induced pore (TIP), primary particle boundary (PPB), and inclusion defects. Among them, inclusion defects are the core problem of powder superalloys [5,6,7,8]. Because of the complex manufacturing process of powder superalloys and the extremely small size of the powder itself, inclusions are inevitable in the actual production process [9]. Whether it is the inconsistency of the properties of the inclusions and the superalloy or the change in the microscopic properties of the surrounding superalloy caused by the inclusion, it will severely affect the mechanical properties of the powder superalloy, especially the low cycle fatigue performance [10]. Therefore, it is necessary to establish a feasible prediction model for the fatigue life of powder superalloys containing inclusions.

Under the framework of fatigue theory, many life prediction methods have been proposed. Chan [11] applied a low-cycle fatigue life model to a Ni-based superalloy and calculated the fatigue crack nucleation cycles of inclusions based on fatigue theory. Hu et al. [12] conducted a low-cycle fatigue experiment of FGH96, established an improved microdamage mechanism Coffin-Manson model and a Bayesian analysis probabilistic model to describe the scattering caused by inclusions. Miao et al. [13] studied the fatigue behavior of FGH96 at high temperatures and explained the large dispersion of fatigue life. Meanwhile, other researchers studied fracture mechanics theory as a tool for predicting life. From the perspective of fracture mechanics, Denda et al. [14] proposed low cycle fatigue lifetime predictions for the inclusion-initiated fatigue and a predictive protocol for determining the inclusion size effect. Using fracture mechanics, Grison et al. [15] presented a probabilistic model of fatigue failure of powder superalloy, which relates to the growth rate of cracks initiated from inclusions, and they discussed the risk of fatigue failure from particles in different positions. Shi et al. [16] proposed a life prediction method based on the fracture mechanics, discussed the parameter sensitivity analysis and the effect on life predictions for the surface crack between different strategies. With increasing experiments and uses of powder superalloys and gradually enriching databases, computer algorithms might develop into one of the mainstream methods for predicting the fatigue life of powder superalloys. Lotfi and Beiss [17] conducted a large number of experiments under different experimental conditions to establish a huge powder superalloy experimental database, built a neural network system using genetic algorithms, and conducted comparative analyses with the database to predict the low cycle fatigue life of powdered superalloys.

However, the mechanism of fatigue crack initiation and propagation and the law of crack initiation have rarely been reported. Liu et al. [18] studied the effects of the Fe-phase and defects on the fatigue life of aluminum alloys through detailed microstructural characterization. The mechanism of fatigue crack initiation and propagation was confirmed by scanning electron microscopy (SEM) and energy-dispersive X-ray spectroscopy (EDS). Fracture surface topography parameters can also provide information for analysis regarding the fatigue loading mode (bending-torsion) [19], strain sequences [20], etc. Using the elastoplastic fracture mechanics (EPFM) method, considering the cumulative damage trend of crack initiation life, Wang et al. [21] studied the fatigue life of short cracks and established a calculation model of fatigue crack initiation size caused by symmetrical cyclic torsional alternating stress, thus laying a foundation for the fatigue life study. Liu and Choi [22] proposed a new method to determine the initial life of a crack by subtracting the predicted crack growth life from the total fatigue life obtained from the experiment. Subsequently, a crack initiation life model was established based on the dislocation model and verified by experiments.

According to the studies on the crack initiation of inclusion powder superalloy FGH96, the crack initiation modes of inclusion powder superalloy FGH96 are mainly as follows: (1) inclusion-matrix interface debonding. This is the main fatigue crack initiation mode in powder superalloys containing inclusions. Irregular shapes of inclusions, uncoordinated deformation of inclusions and matrix materials, chemical reactions between inclusions and matrix materials, etc., making the inclusion-matrix interface very prone to cracks. When a crack occurs at the inclusion-matrix interface, it will quickly spread to the entire interface and extend into the matrix material at 45° to the loading direction. (2) Cracking of the inclusion itself. The inclusion cracks first expand on the equatorial plane perpendicular to the loading direction, and then the internal cracks of the inclusion gradually expand to the inclusion-matrix interface. After the cracks grow to the interface, they will continue to expand along the interface and eventually cause the specimen to fracture. Because of the complex interaction between inclusions and matrix materials and the complex interface stress status, it is still impossible to describe the stress criterion of the interface between inclusions and powder superalloy from a theoretical perspective. The crack initiation life and crack propagation life of the inclusion-matrix interface are only studied at the stage of experimental measurement and statistical estimation, and the problem of inclusion-matrix interface debonding still needs more research. The Beijing Institute of Aeronautical Materials [23] reported that in the powder superalloy FGH96 containing inclusions, the inclusions are regarded as hard inclusions when the material is pure Al_2_O_3_. The fatigue crack of FGH96 with hard inclusions first initiates at the inclusion-matrix interface, then the crack extends to the interface, and finally enters the matrix material to cause fatigue fracture.

Therefore, this research attempts to systematically study the problem of inclusion-matrix interface debonding. First, a finite element model was built, and a fatigue life prediction model based on damage mechanics was developed to calculate the interface crack initiation life. By generating a finite element model to simulate the whole process of interface debonding of the specimen during the loading, the evolution of the stress field of the inclusion-matrix interface was traced. Finally, the effect of the location and shape size of the inclusion on the interface debonding was discussed.

## 2. Construction of Fatigue Life Prediction Model

Based on the thermodynamic theory of damage mechanics [24], the fatigue damage process of metallic materials can be regarded as an irreversible thermodynamic process, which dissipates part of the internal energy in the form of heat. In this process, the Helmholtz free energy *g* is:

(1) $$g=\frac{\sigma_{ij}\varepsilon_{ij}}{2\rho }=\frac{C_{ijkl}\sigma_{kl}\sigma_{ij}}{2\rho (1-D)}$$

where *C_ijkl_* is a fourth-order flexibility tensor, *σ_kl_* is the stress component, and *D* is the damage degree field, which ranges from 0 to 1. When *D* is 0, it means that the material has not started to be damaged, and when it is 1, it means that there is macroscopic crack initiation. The damage characterization parameter *Y* can then be introduced:

(2) $$Y=-\rho \frac{\partial g}{\partial D}=\frac{C_{ijkl}\sigma_{kl}\sigma_{ij}}{2{\left(1-D\right)}^{2}}$$

In the formula, *Y* is related to the damage degree *D*, representing the free energy dissipation caused by the internal damage of the material. During material damage, the damage degree *D* continues to increase, the Helmholtz free energy *g* is continuously dissipated in the form of heat energy, and the material’s external functional force decreases.

Time-dependent damage evolution when cyclic loading is applied can be obtained as follows:

(3) $$\frac{dD}{dN}=aKY_{\max}^{\frac{m}{2}}$$

Among them, *a* and *m* are the damage parameters, and *K* is a parameter introduced to characterize the cyclic load characteristics, known as the cyclic load characteristic value, which is a function of the strain ratio *R*:

(4) $$K=\{\begin{array}{c} 1-R_{+}^{m}\;(\mathrm{under}\;\text{tension-tension}\;\mathrm{load})\\ 1+|R_{-}{|}^{m}\;(\mathrm{under}\;\text{tension-compression}\;\mathrm{load}) \end{array}$$

In the uniaxial strain fatigue test of a nondestructive smooth specimen, there is only one strain component in the loading direction, and the damage characterization parameter *Y* degenerates to:

(5) $$Y_{\max}=\frac{E}{2}\varepsilon_{\max}^{2}$$

By substituting into Equation (3), the damage evolution equation under uniaxial strain fatigue test can be obtained as follows:

(6) $$\frac{dD}{dN}=aK{\left(\frac{E}{2}\right)}^{\frac{m}{2}}\varepsilon_{\max}^{m}$$

By integrating Equation (6) on the load history and taking the logarithm at both ends, a linear relationship can be obtained as follows:

(7) $$\mathrm{lg}N=-m\mathrm{lg}\varepsilon_{\max}+\mathrm{lg}C$$

where the constant $C=\frac{1}{aR{\left(\frac{E}{2}\right)}^{\frac{m}{2}}}$.

Obviously, from Equation (7), the uniaxial strain fatigue test data of the nondestructive (without inclusions) smooth standard specimen under specific conditions can be used to fit the unknown damage parameters *a* and *m*, and then the damage mechanics fatigue life prediction model can be established under the corresponding conditions.

**Figure 1:**
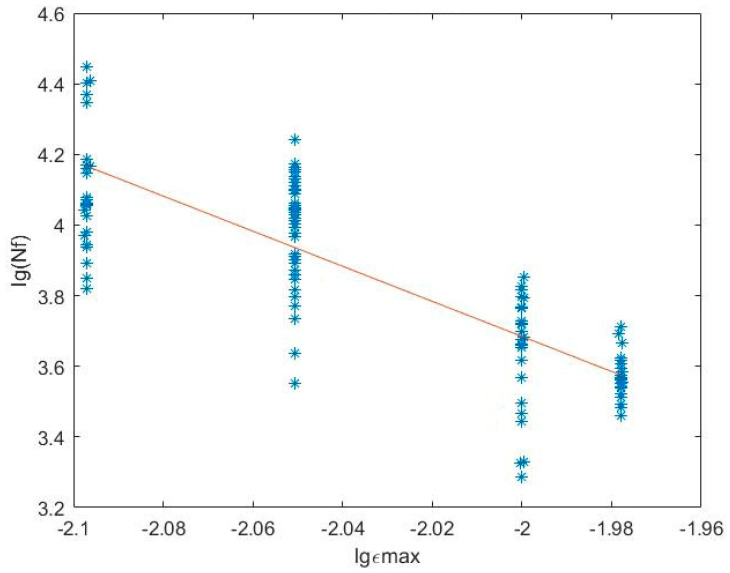
Damage parameter fitting when *R* = 0.05.

The fatigue test of the nondestructive smooth FGH96 specimen was conducted at 530 °C, and the strain ratio *R* = 0.05 with different strain ranges (0.95%, 0.846%, 0.76%, and 1%) on MTS-100kN-7 electro-hydraulic servo fatigue testing machine. The experimental data are listed in the Appendix A. Using MATLAB to fit the uniaxial strain fatigue experimental data, the damage parameters under the corresponding conditions can be obtained. The data fitting results are as follows:

The fitting result shown in Figure 1 indicates that *m* = 4.9684 and *lgC* = −6.2523;

According to the definition of the cyclic load characteristic value:

(8) $$K=1-0.05^{4.9684}\approx 1$$

By substituting the value into $C=\frac{1}{aR{\left(\frac{E}{2}\right)}^{\frac{m}{2}}}$, we obtain *a* = 8.1966 × 10^−7^.

By integrating Formula (3) in the damage interval (*D* = 0–1), the relationship between fatigue life and damage characterization parameter *Y* can be obtained as follows:

(9) $$N=\frac{1}{aKY_{\max}^{\frac{m}{2}}}$$

After adding the damage parameters, *a* and *m* obtained above into Equation (9), the fatigue life prediction model with strain ratio *R* = 0.05 at 530 °C can be obtained as follows:

(10) $$N=\frac{1\times 10^{7}}{8.1966\times K\times Y_{\max}^{2.4842}}$$

## 3. Finite Element Analysis of Interface Debonding and Crack Initiation Life Prediction

For the finite element analysis of the interface debonding problem, it is impossible to directly bond all the faces together using the AGLUE command family in ANSYS (Version 2021 R1, ANSYS Inc., Pittsburgh, PA, USA), and this setting cannot simulate the debonding at a specific position of the interface. Therefore, to simulate the specific process of interfacial debonding, the Debonding module in the contact analysis is innovatively used for finite element modeling.

In ANSYS, Contact Analysis is a common module for solving problems of contact and separation between two objects, and the Debonding module is a submodule in Contact Analysis. It is specially used for studying the contact, bonding, and cracking, and separation of two materials. This module is widely used in the peeling analysis of composite materials. In this study, the basic principles of using the Debonding module to simulate the inclusion-matrix interface can be described as follows:

The interface formed by inclusion and FGH96 matrix is an extremely complex material, which is very thin or even has no thickness [25]. Because of the irregular arrangement of crystals, chemical reactions occurring at the interface during processing, and other reasons, the strength of the inclusion-matrix interface is extremely low, much lower than that of the matrix and inclusion. During loading, tensile cracking is the main failure mode. During the modeling in this study, the elements of two materials adjacent to the inclusion-matrix boundary were set as contact elements, and the stress criterion and the debonding threshold value were set at the contact boundary to simulate the strength of the interface. When the stress value is greater than the debonding threshold value, the inclusion and the matrix will separate from each other. In this manner, the crack propagation along the interface can be simulated. The finite element modeling and analysis of two specimens are briefly described below in Section 3.1, with the interface stress field evolution in Section 3.2.

### 3.1. Interface Debonding Simulation and Crack Initiation Life Prediction

#### 3.1.1. Specimen with Elliptical Subsurface Inclusion

**Figure 2:**
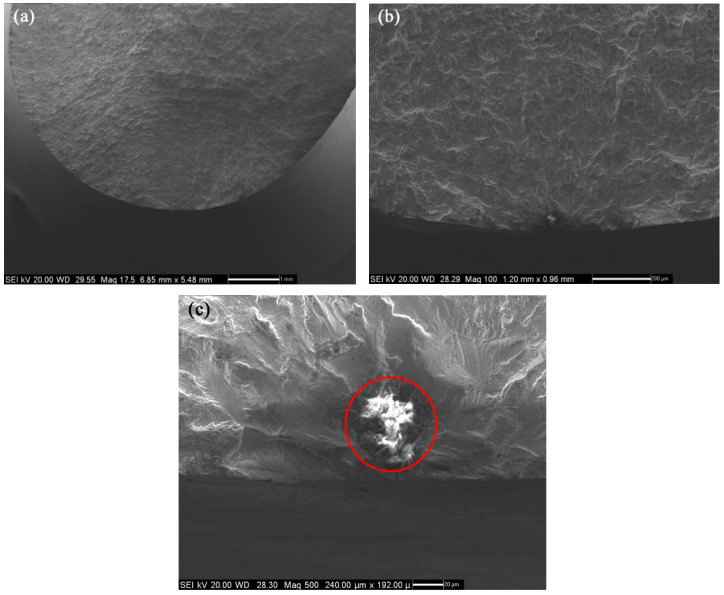
Morphology of the fracture area of specimen No. 1: (**a**) Macro fracture morphology, (**b**) crack source area and crack propagation region, (**c**) crack source area.

**Figure 3:**
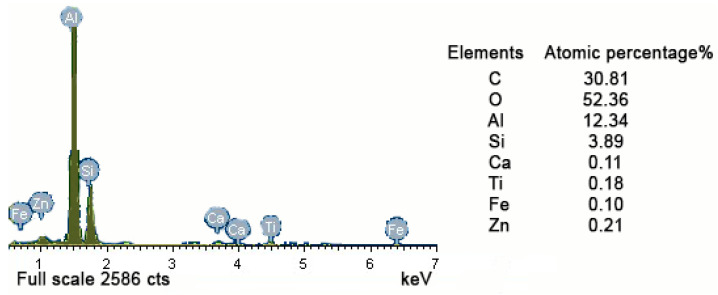
Fracture defect energy spectrum of specimen No. 1.

Table 1 shows the experimental data of a standard uniform cross-sectional cylinder with a cross-sectional radius of 6 mm, named specimen No. 1. The lateral and longitudinal dimensions of the inclusion were measured in the scanning electron micrograph of fracture area of specimen No. 1 shown in Figure 2 below, which are 61 μm and 63 μm, respectively. There is an elliptical inclusion at a distance of 0.04 mm away from the surface of the specimen. It can be inferred from the fracture defect composition of specimen No. 1 shown in Figure 3 that the inclusion material is mainly aluminum oxide, whose elastic modulus is 400 GPa, and the Poisson’s ratio is 0.3. The elastic modulus of matrix material FGH96 is 185.3 GPa, and the Poisson’s ratio is 0.35.

First, the material parameters and element types were set according to the experimental data, and a basic plane model was built. In the contact area between the inclusion and matrix, the copy and partial deletion commands were used to form the contact parts of the two materials. Among them, the contact part of the inclusion was set as the contact surface, and the contact part of the matrix was set as the target surface. In element settings, the matrix and inclusion of the noncontact part were set to PLANE82. The contact surface elements were set to CONTA172, and the target surface elements were set to TARGE169. These two types of two-dimensional (2D) contact elements were used in pairs to define the contact and slip state between deformed surfaces and the tensile stress debonding and shear stress friction slip of contact surfaces.

After the basic size model is ready, element types and material properties were assigned to the inclusion and matrix, and then mesh options were controlled to generate a mesh where 2D quadrilateral meshes were applied. Less dense mesh sizes were used to reduce the calculations computational burden for the meshes at the far end of the inclusion. The total amount of mesh elements for the whole model is 26,989.

**Figure 4:**
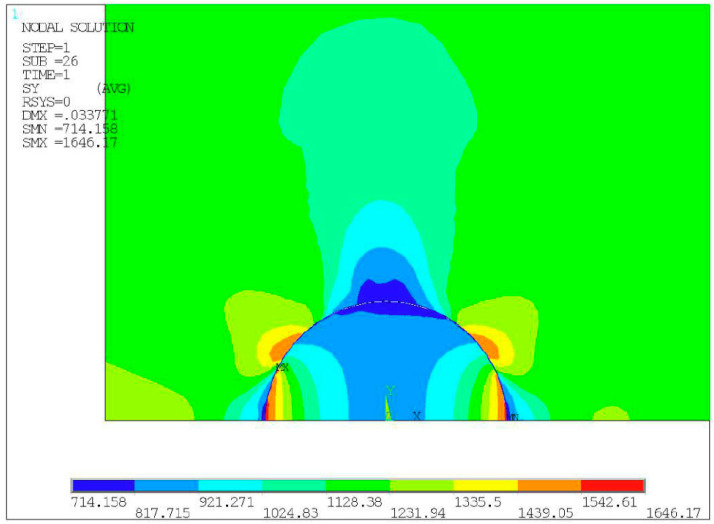
Stress cloud chart result of specimen No. 1.

All the nodes on the element in the contact part were selected, and the material and attributes of the contact element were assigned to complete the preprocessing of the model after the meshing was completed. According to the experimental load conditions, the equivalent displacement of 0.0267 mm was set at the upper end of the model, and a symmetrical boundary condition was set at the lower end of the model. Finally, the stress solution highlighted in the inclusion area is given in Figure 4.

The stress cloud chart shown in Figure 4 shows that with the occurrence of interface debonding at the upper part of the inclusion, the stress between the matrix and inclusion was released, so that this part of the material is in a state of low-stress level, while the stress concentration became very obvious at the crack tip.

**Figure 5:**
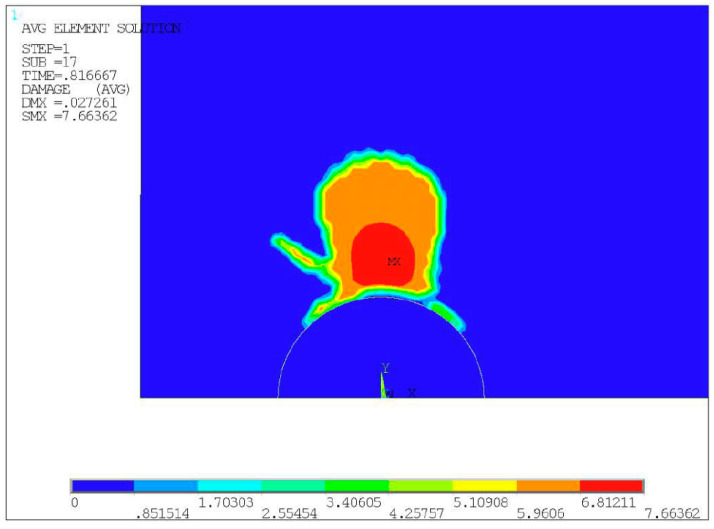
Damage cloud chart of specimen No. 1.

By determining the calculation results of the load substeps just after the crack initiation, and by extracting each strain component of all elements in turn and substituting them into Equation (1), the damage characterization parameter of each element can be obtained, and the damage cloud chart as shown in Figure 5 can be output. The maximum damage characterization parameter *Y_max_* = 7.66362 was then computed for the initiation of the crack in the elements of specimen No. 1, and the interfacial crack initiation life can be determined as 7749 cycles by substituting the *Y_max_* into Equation (10). By comparing with the experimental life of 8125 cycles, the prediction error was claimed as 4.63%, which validated the proposed life prediction method.

#### 3.1.2. Specimen with Semielliptical Surface Inclusion

**Figure 6:**
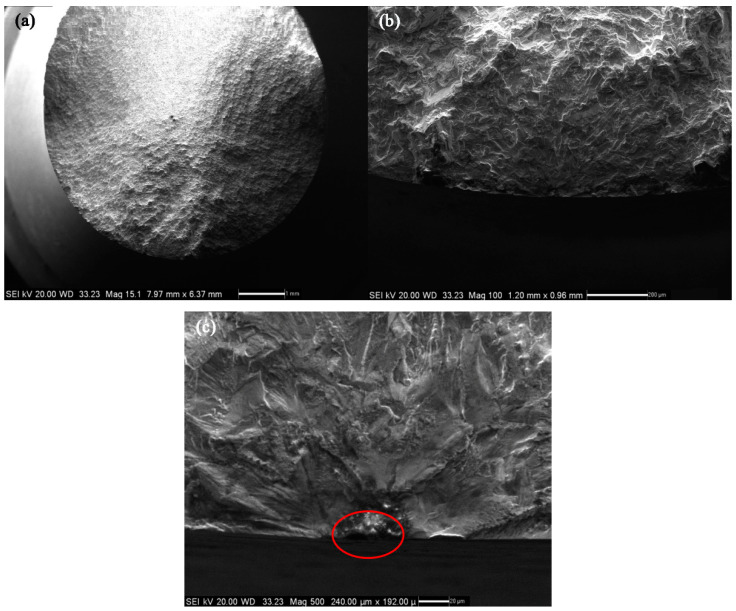
Morphology of fracture zone of specimen No. 2: (**a**) Macrofracture morphology, (**b**) crack source area and crack propagation region, (**c**) crack source area.

**Figure 7:**
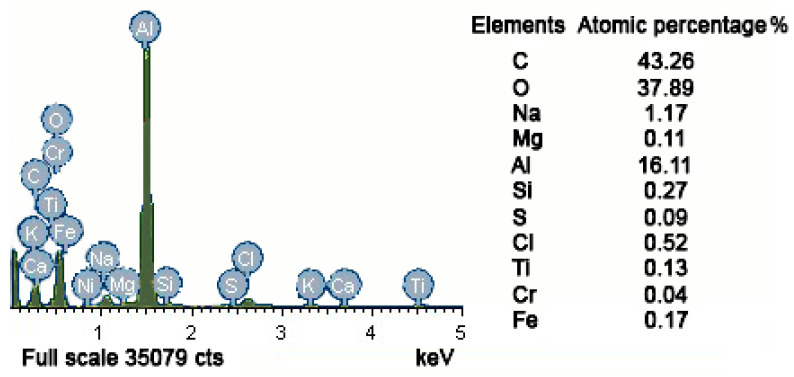
Fracture defect energy spectrum of specimen No. 2.

Test specimen No. 2 is also a cylinder with an equal cross-section. A semielliptical inclusion of 49 μm∙31 μm on the surface of the specimen was measured in this case, as shown in Table 2. The scanning electron micrograph of the fracture area of specimen No. 2 is shown in Figure 6 below. The inclusion material was determined as near-pure aluminum oxide from the fracture defect energy spectrum shown in Figure 7. Its elastic modulus is 400 GPa, and the Poisson’s ratio was set to be 0.3. The material parameters and element type settings are the same as the elements of specimen No. 1 for finite element analysis. Because the overall model is relatively regular and no prominent sharp parts exist, the 2D quadrilateral mesh is still used, where the mapping mesh is used for the inclusion and the matrix far away from the inclusion. To match the elliptical boundary of inclusion, the matrix near the inclusion area was controlled as a quadrilateral, and an automatic mesh division was applied. The total number of mesh elements is around 21,691.

**Figure 8:**
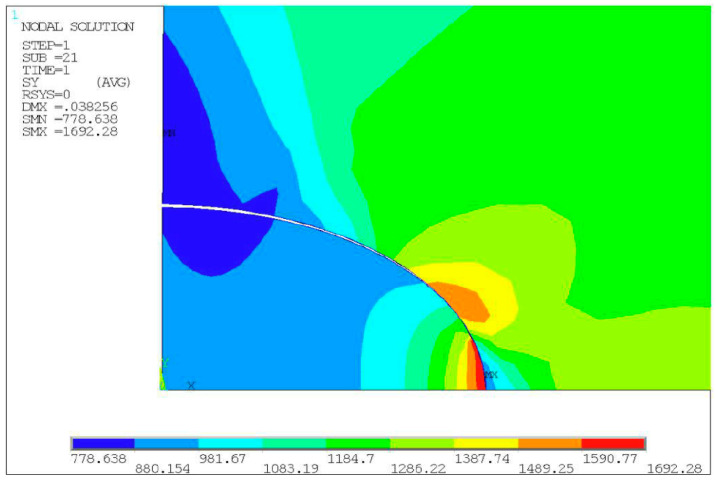
Stress cloud chart result of specimen No. 2.

By analyzing the stress cloud chart shown in Figure 8, it is evident that the maximum stress was calculated at the top of the inclusion when there is no debonding at the matrix-inclusion interface. The stress concentration here, however, leads to the initiation of cracks. Following the occurrence of cracks, the stresses at the interface with the original stress concentration are released, which reduces the stress level at the interface. The stress concentration shifted to the crack tip of the interface between the inclusion and matrix, making it a driving force of crack propagation. As the specimen continues to be loaded, the cracks gradually propagated along the interface and finally entered the matrix material, leading to the fatigue failure of the specimen.

**Figure 9:**
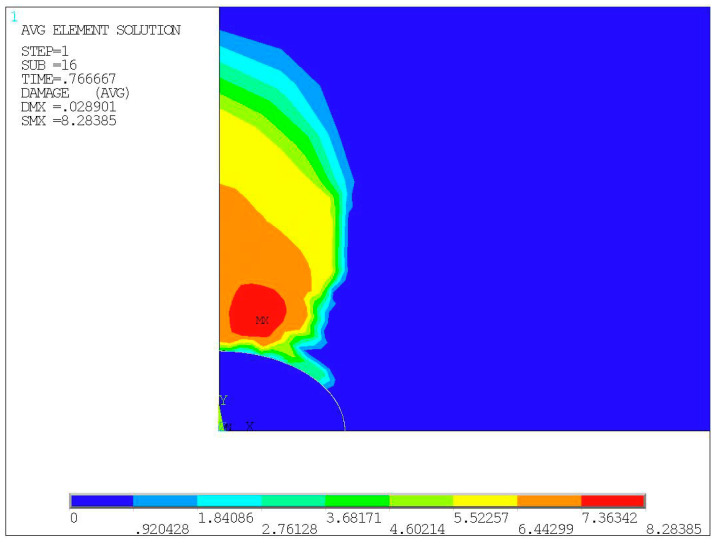
Damage cloud chart of specimen No. 2.

The damage cloud chart shown in Figure 9 can be generated from the calculated results of the load substep at the beginning of crack initiation. By substituting them into Equation (1), the maximum damage characterization parameter *Y_max_* = 8.28385 was obtained. The interfacial crack initiation life was then predicted as 6394 cycles with Equation (10). In comparison with the experimental life of 5820 cycles listed in Table 2, it generates a prediction error of around 9.86%.

**Figure 10:**
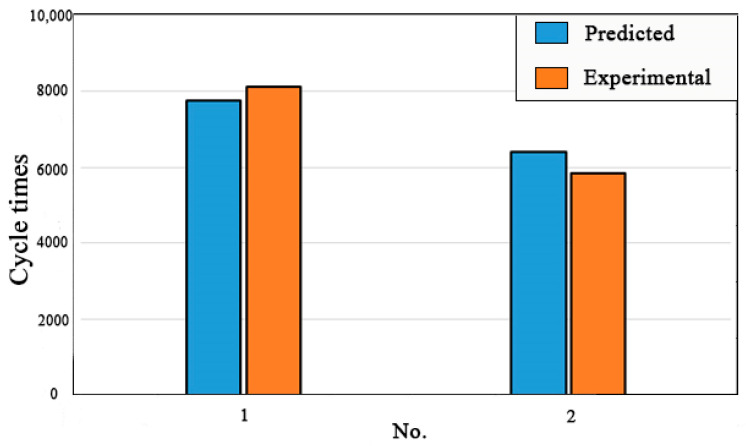
Experimental life vs. Predicted life.

From the analysis described in Section 3.1.1 and Section 3.1.2, it can be concluded that the prediction errors of the interface crack initiation life of the specimens with elliptical subsurface inclusion and specimens with semielliptical surface inclusion are both less than 10%, as shown in Figure 10, and the prediction results are consistent with the test results. The application of CONTA172 and TARGE169 elements proved to effectively simulate the interface debonding between the inclusion and matrix during finite element modeling.

### 3.2. Evolution of Interface Stress Fields

This section gives the evolutional fragments of the interface stress field between the inclusion and matrix. The work was based on test specimens No. 1 and No. 2 mentioned above. The inclusion-matrix interface debonding and crack generation were elaborated by finite element simulation and a brief evaluation of stress distributions around the inclusion-matrix interface during the evolution.

#### 3.2.1. Specimen with Elliptical Subsurface Inclusion

It is observed that the interface stress field will gradually change with the propagation of cracks during the inclusion-matrix interface debonding. First, considering specimen No. 1 in Section 3.1.1, the changes in the stress field of the inclusion-matrix interface before crack initiation and after crack initiation and crack propagation were compared and analyzed.

**Figure 11:**
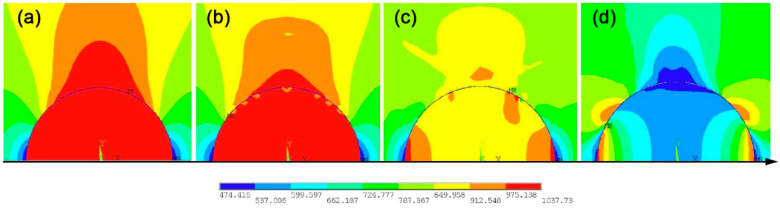
Interface stress field evolution of specimen No. 1: (**a**) before crack initiation, (**b**) crack initiation stage, (**c**) crack propagation stage, (**d**) complete debonding of the interface.

Before the crack initiation, the inclusion and matrix are not separated, and the interface is intact before the crack initiation, as shown in Figure 11a. The stress and strain of the inclusion and matrix material are continuous. The stress concentration is mainly generated in the inclusion and a small part of the matrix material at the upper end of the inclusion (in red part), and the maximum stress is generated at the inclusion-matrix interface.

Many microcracks are generated on the interface between the inclusion and matrix at the same time as the specimen continues to be loaded, as shown in Figure 11b. The effect of stress concentration at the place where the cracks occur is alleviated. While the previous stress concentration disappears, a new stress concentration occurs at the tip of the interface crack, which takes the role of crack propagation driving force.

With the gradual expansion of many microcracks, as shown in Figure 11c, these cracks will be gradually connected together to form larger cracks. The effect of stress concentration of the inclusion and matrix nearby is greatly relieved, but the maximum stress is still at the crack tip, which promotes the cracks to continue to expand.

Figure 11d shows that all the cracks on the interface are finally connected together, and the interface is completely debonded from the upper matrix interface. The cracks begin to enter the matrix, eventually leading to the fatigue fracture of the specimen.

#### 3.2.2. Specimen with Semielliptical Surface Inclusion

**Figure 12:**
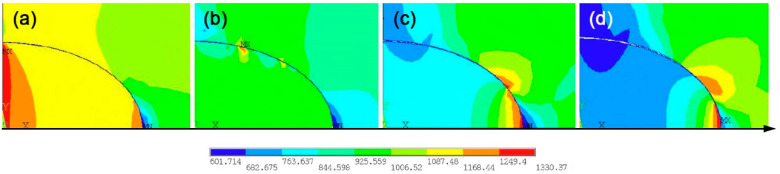
Interface stress field evolution of specimen No. 2: (**a**) Before crack initiation. (**b**) After crack initiation. (**c**) Crack propagation stage. (**d**) Complete debonding of the interface.

The changes in the interface stress field before and after the crack initiation of specimen No. 2 in Section 3.1.2 were observed. The stress concentration was mainly generated at the end of the inclusion close to the surface of the specimen before the crack initiates, as shown in Figure 12a. The maximum stress is located at the place where the inclusion contacts with the matrix with an angle of 90°. This is the stress concentration where the cracks are started. Figure 12b shows the first crack occurrence at the place where the previous concentration takes place as the specimen continues to be loaded. The stress of the inclusion and matrix was then released after the cracks were generated at the interface, and the maximum stress was then observed by computation at the tip of interface cracks.

Finally, the cracks propagate along with the inclusion-matrix interface until the interface is completely separated, as shown in Figure 12c,d, and then the cracks enter the matrix from the interface, rapidly leading to the fatigue fracture of the specimen.

From the analysis in Section 3.2.1 and Section 3.2.2, it can be concluded that cracks generally originate at the place where the stress is the greatest at the inclusion-matrix interface. With the generation of cracks, the stress concentration effect of the inclusion and matrix where the cracks occur will be weakened, and the stress concentration effect at the crack tip will become very prominent. This will further promote the cracks to expand along with the interface and finally make the cracks enter the matrix, leading to fatigue failure.

## 4. Effect of Inclusion Characteristics on Interface Debonding

### 4.1. Effect of Inclusion Location on Crack Initiation

In this section, the effect of the location of inclusion in the specimen on the initiation and propagation of interfacial cracks is discussed. Through finite element software modeling, a circular inclusion with a radius of 0.03 mm was set on the surface of the specimen and subsurface 0.04 mm away from the surface, respectively. The effect of inclusion location on the interface crack initiation was analyzed by comparing the debonding situation of the two interfaces during the loading.

**Figure 13:**
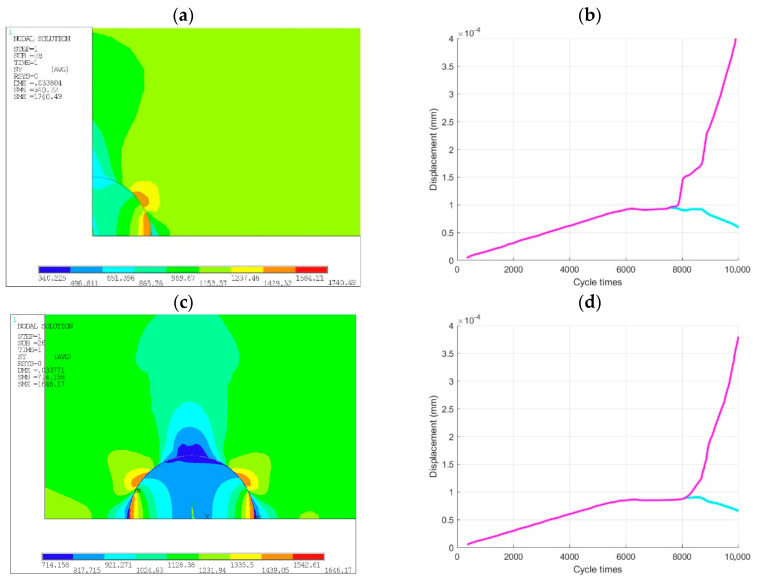
Effect of inclusion location on interface debonding: (**a**,**b**) debonding stress and contact node displacement for surface inclusion. (**c**,**d**) Debonding stress and contact node displacement for surface inclusion.

After the calculation was completed, the two contact nodes on the interface at the top of the inclusion were extracted, and the time-displacement history curve of the contact node is drawn, as shown in Figure 13. The purple curve in the figure is the displacement curve of the node on the interface of the matrix, and the blue curve is the displacement curve of the contact node on the surface of the inclusion.

The time-displacement history graph in Figure 13b,d and stress cloud chart in Figure 13a,c show that when the inclusion is located on the surface of the specimen, fatigue cracks are more likely to initiate on the interface between the inclusion and matrix compared with that in the subsurface inclusion case. Furthermore, under the same load conditions, when the surface inclusion cracks, the maximum stress generated by the stress concentration at the crack tip is also greater. Thus, the interfacial crack propagation speed of the surface inclusion is also greater than that of the subsurface inclusion, and thus the fatigue life of the powder superalloy with surface inclusion is less than that of the powder superalloy with subsurface inclusion.

### 4.2. Effect of Inclusion Shape Size on Crack Initiation

In this section, the effect of inclusion shape size on the crack initiation and propagation at the inclusion-matrix interface is discussed. By using the finite element modeling method, a hard elliptical inclusion was set on the subsurface of the matrix, and the size ratio c/a of the inclusions was set to 0.58, 1, and 2 in turn with the other experimental conditions unchanged. The effect of inclusion shape on the initiation of interface cracks was compared and analyzed.

**Figure 14:**
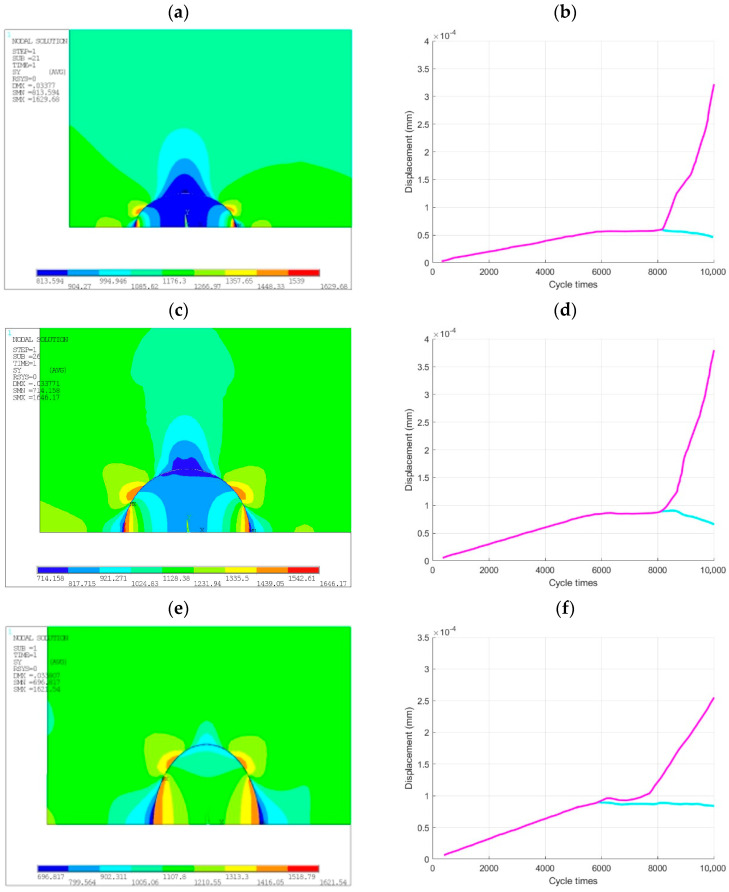
Effect of inclusion shape on interface debonding: (**a**,**b**) debonding stress and contact node displacement for c/a = 0.58. (**c**,**d**) Debonding stress and contact node displacement for c/a = 1. (**e**,**f**) Debonding stress and contact node displacement for c/a = 2.

By observing the stress cloud diagram and the time-displacement history curve of the contact node, as shown in Figure 14, it is evident that when the inclusion is located at the same position of the matrix, the maximum stress is always located at the tip of the interface crack. Regardless of the eccentricity of the inclusion, the interface cracks always start at the top of the inclusion, then propagate along the interface, and finally enter the matrix. The displacement-time history graph shows that the larger the size of the inclusion in the loading direction, the easier the crack imitation at the inclusion-matrix interface, and the shorter the fatigue life. When the size of the inclusion in the loading direction is large, and the size perpendicular to the loading direction is small, the interface is prone to crack. When cracks propagate along the interface, due to the small lateral size of the inclusion, the interface with a smaller angle to the loading direction is easy to withstand a high shear stress, and the failure mode of the interface gradually transits from tensile cracking to shear cracking. Meanwhile, the interface cracks can easily enter the matrix along the slip line of 45° to the loading direction, leading to a rapid fatigue fracture of the specimen.

## 5. Conclusions

This study features the initiation and propagation of fatigue crack initiated from inclusions in powder superalloy material FGH96. Contact analysis was used to simulate the inclusion-matrix interface debonding of the powder superalloy with inclusion during a finite element simulation. The fatigue life prediction model of the powder superalloy with inclusion, which is based on damage mechanics, was applied to calculate the interface crack initiation life with elliptical subsurface and semielliptical surface inclusions. The evolution of stress field during the interface crack propagation was analyzed based on the proposed interface debonding modeling and computation. In addition, the effect of the location and shape size of inclusion on the interface debonding was also evaluated. The following conclusions are drawn:

(1) The use of CONTA172 and TARGE169 in the finite element modeling can effectively simulate the interface debonding between the inclusion and matrix. The prediction results are consistent with the test results, and the prediction errors of interface crack initiation life are less than 10%.

(2) Inclusion-matrix interface cracks are first initiated at the place of stress concentration in the form of tensile cracking, and then the stress concentration effect is transferred to the crack tip; the cracks gradually propagate along the interface; and they finally enter the matrix, leading to fatigue fracture.

(3) The fatigue life of powder superalloy with a surface inclusion is less than that of powder superalloy with a subsurface inclusion under the same loading conditions due to greater concentrated stress at the crack tip.

(4) The debonding mode of the interface is related to the orientation of the interface and loading. When the angle between the interface and loading direction is large, the interface is mainly destroyed in the form of tensile cracking. When it is small, the interface has mainly shear cracking.

## Figures and Tables

**Table 1:** Experimental data of specimen No. 1.

Strain Ratio	Strain Range (%)	Cycle Times	Diameter (mm)	Main Components of Inclusion	Inclusion Shape	2a (μm)	2c (μm)	Distance between Inclusion & Surface (mm)
0.05	0.846	8125	6	Al_2_O_3_	Ellipse	61	63	0.04

**Table 2:** Experimental Data of Specimen No. 2.

Strain Ratio	Strain Range (%)	Cycle Times	Diameter (mm)	Main Components of Inclusion	Inclusion Shape	2a (μm)	2c (μm)	Distance between Inclusion& Surface (mm)
0.05	0.95	5820	6	Al_2_O_3_	Semiellipse	49	31	0

## Data Availability

3rd Party Data. Restrictions apply to the availability of these data. Data was obtained from AECC Beijing Institute of Aeronautical Materials and are available from the authors with the permission of AECC Beijing Institute of Aeronautical Materials.

## Appendix A

**Table A1:** Fatigue test data of T = 530 °C, R = 0.05, strain range 0.95%.

No.	Max Strain ε_max_	Cycle Times *N_f_*	No.	Max Strain ε_max_	Cycle Times *N_f_*	No.	Max Strain ε_max_	Cycle Times *N_f_*
1	0.01001	2141	11	0.01	5267	21	0.01	2770
2	0.01	4135	12	0.01	6724	22	0.01	3700
3	0.01	6268	13	0.01	4560	23	0.01	4988
4	0.01	5232	14	0.01	3142	24	0.01	5842
5	0.01	4610	15	0.01	5210	25	0.01	4745
6	0.01	5333	16	0.01	5210	26	0.01	4490
7	0.01	4732	17	0.01	4756	27	0.01	5820
8	0.01001	7146	18	0.01	1937	28	0.01001	4823
9	0.01	6535	19	0.01	2939	29	0.01001	6235
10	0.00999	2114	20	0.00991	2880	-	-	-

**Table A2:** Fatigue test data of T = 530 °C, R = 0.05, strain range 0.846%.

No.	Max Strain ε_max_	Cycle Times *N_f_*	No.	Max Strain ε_max_	Cycle Times *N_f_*	No.	Max Strain ε_max_	Cycle Times *N_f_*
30	0.0089	8301	46	0.0089	6567	61	0.0089	12,249
31	0.0089	10,454	47	0.0089	14,521	62	0.0089	13,716
32	0.0089	11,376	48	0.0089	8125	63	0.0089	14,174
33	0.0089	13,500	49	0.0089	12,616	64	0.0089	14,856
34	0.0089	12,219	50	0.0089	9254	65	0.0089	11,188
35	0.0089	9285	51	0.0089	13,550	66	0.0089	13,672
36	0.0089	12,504	52	0.0089	9857	67	0.0089	10,308
37	0.0089	9509	53	0.0089	7427	68	0.0089	10,726
38	0.0089	11,116	54	0.0089	7771	69	0.0089	17,389
39	0.0089	7962	55	0.0089	10,289	70	0.0089	10,084
40	0.0089	11,518	56	0.0089	10,877	71	0.0089	3568
41	0.0089	7209	57	0.0089	5911	72	0.0089	4330
42	0.0089	13,182	58	0.0089	6276	73	0.0089	6996
43	0.0089	6544	59	0.0089	9246	74	0.0089	14,295
44	0.0089	12,900	60	0.0089	13,204	75	0.0089	5434
45	0.0089	11,031	-	-	-	-	-	-

**Table A3:** Fatigue test data of T = 530 °C, R = 0.05, strain range 0.76%.

No.	Max Strain ε_max_	Cycle Times *N_f_*	No.	Max Strain ε_max_	Cycle Times *N_f_*	No.	Max Strain ε_max_	Cycle Times *N_f_*
76	0.008	9584	85	0.008	6595	93	0.00799	10,985
77	0.008	22,138	86	0.008	23,388	94	0.008	11,970
78	0.008	10,633	87	0.008	7064	95	0.008	14,817
79	0.008	25,297	88	0.00799	9360	96	0.008	14,042
80	0.008	11,755	89	0.008	8765	97	0.008	14,443
81	0.008	11,522	90	0.008	11,387	98	0.008	15,368
82	0.008	28,084	91	0.008	8689	99	0.008	11,467
83	0.008	15332	92	0.00801	25,615	100	0.00801	14,666
84	0.008	7801	-	-	-	-	-	--

**Table A4:** Fatigue test data of T = 530 °C, R = 0.05, strain range 1%.

No.	Max Strain ε_max_	Cycle Times *N_f_*	No.	Max Strain ε_max_	Cycle Times *N_f_*	No.	Max Strain ε_max_	Cycle Times *N_f_*
101	0.01052	3663	110	0.01052	3108	118	0.01052	3654
102	0.01052	3693	111	0.01052	3050	119	0.01052	4196
103	0.01052	3768	112	0.01051	4938	120	0.01052	4217
104	0.01052	3255	113	0.01053	3589	121	0.01052	4149
105	0.01052	3935	114	0.01052	5166	122	0.01052	3475
106	0.01052	3564	115	0.01053	4649	123	0.01053	3843
107	0.01052	3449	116	0.01052	3742	124	0.01052	4051
108	0.01052	3334	117	0.01052	3916	125	0.01052	2883
109	0.01052	3472	-	-	-	-	-	-
